# The Gut Microbiota-Produced Indole-3-Propionic Acid Confers the Antihyperlipidemic Effect of Mulberry-Derived 1-Deoxynojirimycin

**DOI:** 10.1128/mSystems.00313-20

**Published:** 2020-10-06

**Authors:** Yougui Li, Wenyi Xu, Fang Zhang, Shi Zhong, Yuqing Sun, Jinxi Huo, Jianxun Zhu, Chongming Wu

**Affiliations:** a Sericultural Research Institute, Zhejiang Academy of Agricultural Science, Hangzhou, People’s Republic of China; b Beijing Advanced Innovation Center for Food Nutrition and Human Health, China Agricultural University, Beijing, People’s Republic of China; c Pharmacology and Toxicology Research Center, Institute of Medicinal Plant Development, Chinese Academy of Medical Sciences & Peking Union Medical College, Beijing, People’s Republic of China; Vall d’Hebron Research Institute (Ed. Mediterranea)

**Keywords:** 1-deoxynojirimycin, gender disparity, gut microbiota, hyperlipidemia, indole-3-propionic acid

## Abstract

Hyperlipidemia is a worldwide epidemic with an obvious gender disparity in incidence. Modulations on gut microbiota by traditional Chinese medicines (TCM) are emerging as a potential rationale governing the profitable effects of drugs on hyperlipidemia. However, it is unclear how gut microbes regulate the progression of hyperlipidemia. Here, we found that mulberry leaf extract (MLE) and its active component 1-deoxynojirimycin (DNJ) diminished hyperglycemia and hypertriglyceridemia with similar efficacy in male and female mice but preferentially alleviated hypercholesterolemia in female mice. Further investigations showed that DNJ sex-specifically downregulated the expression of lipogenic genes, especially cholesterol-biosynthetic genes. Oral administration of DNJ imposed more profound modulation on gut microbiota in female mice than in male ones, as estimated by 16S rRNA metatranscriptomic analysis. DNJ markedly enriched Akkermansia and Clostridium group XIVa and promoted the production of indole-3-propionic acid (IPA) in a sexually dimorphic way. Importantly, IPA tightly associates with the antihyperlipidemic effect of DNJ and exhibited a potent lipid-lowering effect both *in vitro* and *in vivo*. Together, our results have established a regulatory mechanism by which DNJ sex-specifically improves hyperlipidemia, offering an in-depth theoretical basis for therapeutic exploitation of DNJ as a sex-specific intervention against hyperlipidemia.

**IMPORTANCE** Hyperlipidemia has been intensively focused on by researchers around the world owing to its major contribution to cardiovascular diseases. Various evidence reveals that women are more susceptible than male counterparts to dyslipidemia, making sex-dependent therapeutic strategies and drugs urgently needed. In the present work, we demonstrate that DNJ, the main active component of mulberry leaves, exerts an obvious female-preferential antihyperlipidemic effect through specifically enriching *Akkermansia* and *Clostridium* XIVa and elevating an active microbial metabolite, indole-3-propionic acid (IPA), in female mice. Moreover, we have corroborated the potent lipid-lowering efficacy of IPA both *in vitro* and *in vivo*. These findings not only indicate a potential mechanism by which gut microbes and their metabolites confer the beneficial role of DNJ in ameliorating hyperlipidemia but also provide an in-depth theoretical basis for therapeutic exploitation of DNJ as a female-specific intervention against hyperlipidemia.

## Abstract (precis)

Hyperlipidemia has been intensively focused on by researchers around the world owing to its major contribution to cardiovascular diseases. Various evidence reveals that women are more susceptible than male counterparts to dyslipidemia, making sex-dependent therapeutic strategies and drugs urgently needed. In the present work, we demonstrate that DNJ, the main active component of mulberry leaves, exerts an obvious female-preferential antihyperlipidemic effect through specifically enriching *Akkermansia* and *Clostridium* XIVa and elevating an active microbial metabolite, indole-3-propionic acid (IPA), in female mice. Moreover, we have corroborated the potent lipid-lowering efficacy of IPA both *in vitro* and *in vivo*. These findings not only indicate a potential mechanism by which gut microbes and their metabolites confer the beneficial role of DNJ in ameliorating hyperlipidemia but also provide an in-depth theoretical basis for therapeutic exploitation of DNJ as a female-specific intervention against hyperlipidemia.

## INTRODUCTION

Hyperlipidemia is a major cause of atherosclerotic cardiovascular diseases, which have become prevalent all over the world. Recent clinical investigations have revealed that there exist apparent gender disparities in the incidence of hyperlipidemia; that is, women are more susceptible than their male counterparts to dyslipidemia (1, 2). In particular, women usually have higher low-density lipoprotein (LDL) levels than men (3, 4) and are likely to discontinue lipid-lowering therapies (1, 5). Ultimately, more women are left with uncontrolled hyperlipidemia by current medication (6–8). Therefore, discovering novel female-preferential therapeutic methods is urgently needed.

The gut microbiota, the multifaceted commensal community in human beings and animals, plays an indispensable role in maintaining host homeostasis (9). Dysbiosis of gut flora leads to various metabolic diseases, such as obesity, hyperlipidemia, and type 2 diabetes (10). A growing body of evidence has demonstrated that the gut microbiota is crucial for the maintenance of human health (11) and plays a crucial role in the beneficial effects of various nutrients and drugs (12–15). Recent investigations have shown that gender differences in the gut microbial community and its metabolites are novel causes for the sex bias in immunity response (16, 17) and diet-induced steatosis (18), suggesting that the sex-specific regulation of gut microbiota may be a useful strategy for the development of next-generation therapeutic drugs.

Mulberry leaves have been broadly used as functional foods (as food-grade powder or tea) and traditional Chinese medicine for the prevention and treatment of diabetes and hyperlipidemia (19). 1-Deoxynojirimycin (DNJ) is a major active component of mulberry leaves with well-documented diabetes-ameliorating effects (20). The DNJ-rich mulberry alkaloid tablet is now an approved medicine for type 2 diabetes. Besides potent α-glucosidase inhibitory activity (21), DNJ is also an adequate antihyperlipidemic agent (22, 23). We have previously reported that the antihyperlipidemic effect of DNJ exhibited obvious sex differences and that this sex-specific effect was associated with a sex-specific modulation of gut microbes, preferentially in females (24). However, the particular intestinal bacteria and the main active microbial metabolites are still unknown. For more insights into therapeutic interventions, further investigations are awaited to corroborate the female-specific effect of DNJ on hyperlipidemia, and the precise mechanism governing the sexual dimorphism remains to be elucidated.

In the present study, we demonstrate that DNJ is a potent sex-specific antihypercholesterolemic agent. Supplementation with DNJ sex-specifically modulates gut microbes (e.g., Akkermansia and Clostridium cluster XIVa) and promotes the production of indole-3-propionic acid (IPA) preferentially in females. Importantly, IPA tightly associates with the antihyperlipidemic effect of DNJ and exhibited a potent lipid-lowering effect both *in vitro* and *in vivo*. Therefore, our studies have identified a regulatory mechanism by which DNJ preferentially ameliorates hyperlipidemia in females via modulating certain taxa and metabolite IPA, providing a promising therapeutic strategy for women suffering from hyperlipidemia.

## RESULTS

### Mulberry leaf extract and DNJ ameliorate hyperlipidemia in a sexually dimorphic way.

**FIG 1:**
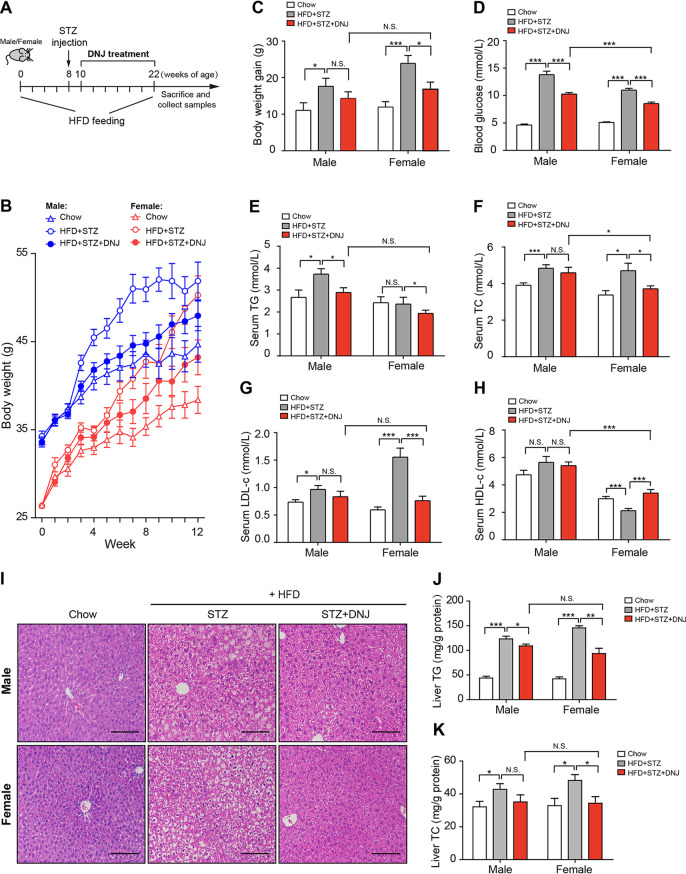
DNJ sex-specifically ameliorates hypercholesterolemia in female diabetic mice. (A) Diagram of the animal experiment design. (B) Bodyweight curve. (C) Bodyweight gain. (D) Fasting blood glucose level. (E to H) Serum levels of TG, TC, LDL-c, and HDL-c. (I) Representative hematoxylin and eosin (H&E) staining images of the liver. Bar, 100 μm. (J to K) Liver content of TG and TC. The chow group was fed with normal chow, the STZ group was fed with an HFD and received a single dose (30 mg/kg) of streptozotocin (STZ) intraperitoneally (i.p.), and the DNJ group was treated like the STZ group and administered DNJ (50 mg/kg) daily by oral gavage. *N* = 10/9 in each male/female group, respectively. Data are presented as mean ± standard error of the mean (SEM). *, *P < *0.05; **, *P < *0.01; ***, *P < *0.001; N.S., not significant.

Given the dual protective effects of DNJ against hyperglycemia and hyperlipidemia, we established animal models suffering from both hyperlipidemia and hyperglycemia by high-fat diet (HFD) feeding combined with a low-dose streptozotocin (STZ) injection (30 mg/kg) (see Fig. S1A in the supplemental material). During the HFD feeding period, ingestion of mulberry leaf extract (MLE) significantly decreased bodyweight gain and alleviated hyperglycemia and hypertriglyceridemia in both male and female mice in a dosage-dependent manner (Fig. S1A to C). However, MLE showed little impact on serum levels of total cholesterol (TC), low-density lipoprotein cholesterol (LDL-c), and high-density lipoprotein cholesterol (HDL-c) in male mice, regardless of the dosage, whereas it substantially restored levels in female mice (Fig. S1D to F), revealing an obvious female-preferential antihypercholesterolemic action. As the main active component of mulberry leaves, oral administration of DNJ (50 mg/kg) reduced bodyweight gain in both genders of mice, yet with a significant influence on female animals (Fig. 1A to C and Table 1). Consistent with the effects of mulberry leaves, serum levels of glucose and TG were dramatically decreased by DNJ treatment in both genders of mice (Fig. 1D and E and Table 1), whereas TC, LDL-c, and HDL-c were restored to a normal range exclusively in female mice, corroborating the sex-specific cholesterol-lowering effects of MLE (Fig. 1F to H and Table 1). Moreover, either MLE or DNJ obviously relieved liver steatosis in both genders, while it exhibited more profound efficiency in female mice (Fig. 1I to K, Table 1 and Fig. S1H-J). Together, these results demonstrate that mulberry leaves and their active component DNJ exert a sex-specific protective effect on hyperlipidemia, especially hypercholesterolemia in diabetic mice.

**TABLE 1:** Statistics showing the influences of DNJ on indicated physiological indicators and expression of hepatic genes involved in lipogenesis between male and female mice

Parameter	*P* value for:				DNJ vs HFD (%)		*P* value^*a*^
	Male		Female				
	HFD vs chow	DNJ vs HFD	HFD vs chow	DNJ vs HFD	Male	Female	
Blood parameters							
Body wt gain	0.0325	0.2624	0.0004	0.0244	−18.73	−31.17	0.1449
Glucose	0.0000	0.0001	0.0000	0.0000	−25.56	−22.35	0.0067
TG	0.0170	0.0148	0.3274	0.0432	−27.64	−17.25	0.0571
TC	0.0008	0.4844	0.0123	0.0384	−5.21	−21.04	0.0451
LDL-c	0.0131	0.3193	0.0000	0.0004	−12.23	−52.69	0.0000
HDL-c	0.2288	0.6572	0.0009	0.0006	−3.55	54.79	0.0008
Liver TG	0.0000	0.0257	0.0000	0.0059	−11.50	−28.23	0.0000
Liver TC	0.0405	0.1371	0.0167	0.0209	−17.86	−28.75	0.3072
Gene expression							
*SREBP1c*	0.0060	0.0017	0.0000	0.0020	−42.71	−41.26	0.7612
*FAS*	0.0000	0.0577	0.0000	0.0013	−33.43	−47.62	0.0650
*ACC*	0.0015	0.0545	0.0000	0.0191	−49.31	−36.85	0.1717
*GPAT*	0.0188	0.3929	0.0000	0.0118	−21.03	−39.74	0.0615
*SCD*	0.0441	0.2641	0.0000	0.0052	−34.38	−50.35	0.0542
*ChREBP*	0.0013	0.1635	0.0002	0.0174	−23.06	−36.64	0.2003
*SREBP2*	0.0000	0.0610	0.0000	0.0029	−21.30	−30.18	0.0781
*HMGR*	0.0000	0.8533	0.0000	0.0143	−1.39	−30.07	0.0000
Taxonomic abundances							
*Clostridium* XlVa	0.0544	0.7467	0.0488	0.0157	−4.77	122.52	0.0037
*Akkermansia*	0.3524	0.4577	0.2083	0.0733	−73.21	9,907.74	0.0514
Metabolite signals							
3-Indolepropionic acid	0.0402	0.0282	0.1744	0.0023	159.3053	650.7457	0.0033
*N*-Acetyl-l-methionine	0.0491	0.8052	0.1023	0.1326	6.7713	−53.1748	0.0178
Hydroxyisobutyrate	0.0029	0.4263	0.0021	0.0604	13.2269	25.5371	0.5223

a DNJ vs HFD between males and females.

10.1128/mSystems.00313-20.3 FIG S1 Effects of mulberry leaf extract (MLE) on blood glucose, lipid profile, and hepatic gene expression in type 2 diabetic mice. (A) Diagram of the animal experiment design. (B) Bodyweight gain. (C) Fasting blood glucose level. (D to G) Serum levels of total triglyceride (TG), total cholesterol (TC), low-density lipoprotein cholesterol (LDL-c), and high-density lipoprotein cholesterol (HDL-c). (H) Representative hematoxylin and eosin (H&E) staining images of the liver. Bar, 200 μm. (I to J) Liver contents of TG and TC. (K) Expression profiles of hepatic genes involved in lipogenesis in response to MLE treatment. *N* = 10 in each group. Data are presented as mean ± standard error of the mean (SEM). *, *P < *0.05; **, *P < *0.01; ***, *P < *0.001; N.S., not significant. Download FIG S1, TIF file, 1.5 MB. Copyright © 2020 Li et al. 2020 Li et al. This content is distributed under the terms of the Creative Commons Attribution 4.0 International license.

### DNJ especially inhibits the expression of lipogenic genes.

**FIG 2:**
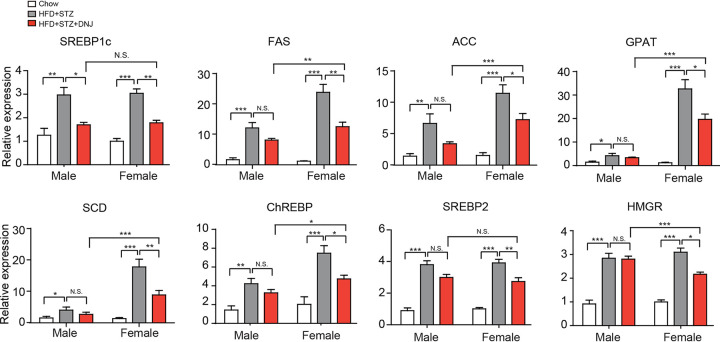
DNJ decreases transcription of lipogenic genes in mice liver. *N* = 10/9 in each male/female group, respectively. Data are presented as mean ± SEM. *, *P < *0.05; **, *P < *0.01; ***, *P < *0.001.

To illuminate the molecular mechanism underpinning the hyperlipidemia-ameliorating role of DNJ, we further examined the impact of DNJ on hepatic genes that are involved in lipid synthesis (*SREBP1c*, *FAS*, *ACC*, *GPAT*, *SCD*, *ChREBP*, *SREBP2*, and *HMGR*), transport (*FATP*, *FABP*, *LXR*, and *CD36*) and oxidation (*CPT1*, *PPARα*, *AMPK*, and *ACO*). Compared with the normal control mice, mice that received gavage of DNJ alone showed decreased transcription of lipogenic genes (Fig. 2 and Table 1), with no significant effects on genes participating in lipid transport (except *CD36*) and oxidation (see Fig. S2 in the supplemental material, suggesting that DNJ may prevent hyperlipidemia via inhibiting lipid biosynthesis. For the key genes involved in fatty acid biosynthesis, including *SREBP1c*, *FAS*, and *ACC*, treatment with DNJ robustly decreased mRNA levels in both male and female mice, with higher efficacy in the latter (Fig. 2). For other fatty acid biosynthetic genes, such as *GPAT*, *SCD*, and *ChREBP*, DNJ only significantly suppressed transcription in female mice. Blood lipid measurement showed that DNJ only decreased TC and LDL-c levels in female mice. Accordingly, the expression of two key cholesterol biosynthetic genes, *SREBP2* and *HMGR*, was only reduced by DNJ in female mice but not in male ones (Fig. 2 and Table 1). Besides, the aforementioned hepatic genes in mice with MLE treatment had similar expression profiles (Fig. S1K). These results indicate that the sex-dependent antihyperlipidemic effects of DNJ may mainly be due to the inhibition in the expression of hepatic lipogenic genes.

10.1128/mSystems.00313-20.4 FIG S2 Effect of DNJ on genes associated with fatty acid uptake, transport, and β-oxidation. The relative mRNA levels of genes were determined by real-time PCR. *N* = 10/9 in each male/female group, respectively. Data are expressed as mean ± SEM. *, *P < *0.05; **, *P < *0.01, ***, *P < *0.001. Download FIG S2, TIF file, 0.4 MB. Copyright © 2020 Li et al. 2020 Li et al. This content is distributed under the terms of the Creative Commons Attribution 4.0 International license.

### DNJ alters gut microbial structure in a sex-specific manner.

Recent investigations showed sex-specific modulation of gut microbiota, which may shed new light on the development of novel precise drugs for a particular gender (18). To get a comprehensive understanding of the regulation on gut microbiome by DNJ, we analyzed the gut microbial community by 16S rRNA gene pyrosequencing to reveal the response of total gut microbes to DNJ treatment. 16S rRNA gene-based metagenomics analysis exhibited that samples in the same gender group tended to cluster together and, but there are still some overlaps between groups. The animals treated with HFD and STZ (HFD + STZ) separated from the normal control in both genders, as revealed by principal-coordinate analysis (PCoA) (see Fig. S3A in the supplemental material) and nonmetric multidimensional scaling (NMDS) (Fig. S3B). Oral administration of DNJ (HFD + STZ + DNJ) did not significantly change the microbial community compared to that in the HFD + STZ group in both genders (Fig. S3A to C). The relative abundance of *Clostridium* XIVa was increased by DNJ in both male and female mice (Fig. S3E), while that of *Akkermansia* was only moderately increased by DNJ in female animals (Fig. S3D). These results revealed a slight sex-specific trend, but the impact of DNJ on the gut microbiota was not distinct between different genders, which may be due to the fact DNA-based metagenomics does not distinguish the active from inactive members of a microbiome and thus cannot discriminate those that are contributing to observed functions from those that are merely present.

10.1128/mSystems.00313-20.5 FIG S3 16S rRNA gene-based metagenomic analysis of gut microbiota. (A and B) Principal-coordinate analysis (PCoA) and nonmetric multidimensional scaling analysis (NMDS). (C) The accumulative abundance of the top 31 genera among the different groups. (D and E) Box plots of the relative abundance of *Akkermansia* and *Clostridium* XIVa species. *N* = 10/9 in each male/female group, respectively. Data are presented as mean ± SEM; *, *P < *0.05. Download FIG S3, TIF file, 1.6 MB. Copyright © 2020 Li et al. 2020 Li et al. This content is distributed under the terms of the Creative Commons Attribution 4.0 International license.

**FIG 3:**
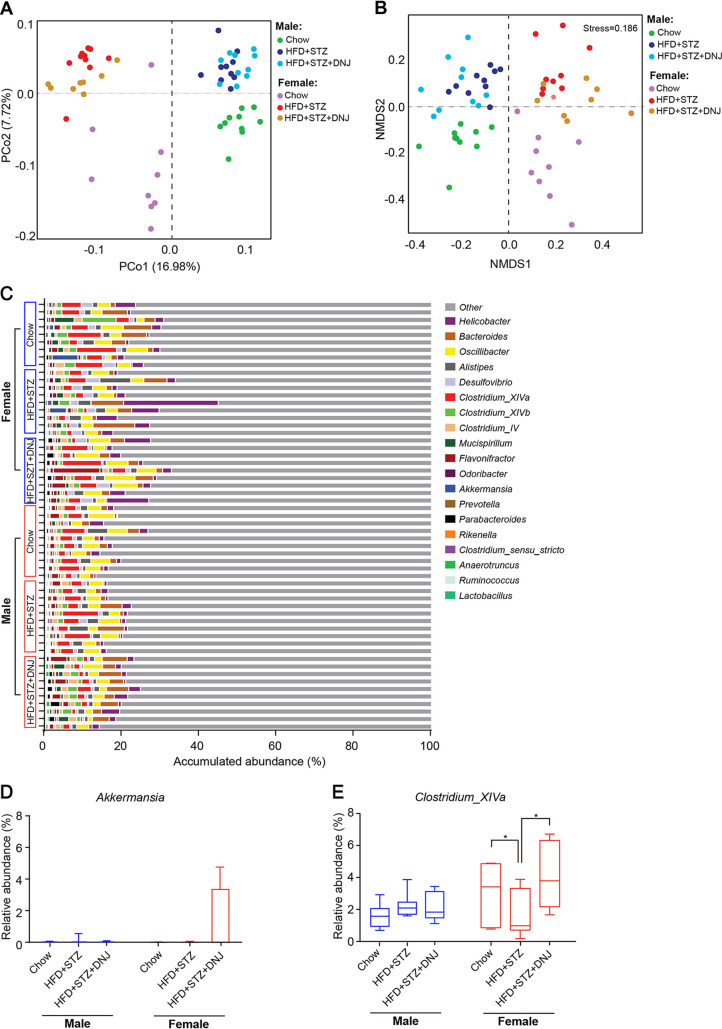
16S rRNA-based metatranscriptomic analysis of gut microbiota. (A and B) Principle coordination analysis (PCoA) and nonmetric multidimensional scaling analysis (NMDS). (C) The accumulative abundance of the top 20 genera among the different groups. (D) Box plots of the relative abundance of *Akkermansia* and *Clostridium* XIVa taxa. *N* = 10/9 in each male/female group, respectively. Data are presented as mean ± SEM. *, *P < *0.05.

To surmount the limitation of 16S rRNA gene-based metagenomics, we further performed 16S rRNA pyrosequencing to specifically reveal the response of live microbes. Male and female samples completely separated, and samples belonging to HFD + STZ-treated groups shifted from the normal control ones in both sexes (Fig. 3A and B). Oral administration of DNJ significantly shifted the live gut microbial community to the normal group in female mice but not in male ones (Fig. 3A and B; *P < *0.001 in the “adonis” test for PCoA analysis). Accordingly, DNJ markedly altered the composition of the live intestinal microbiota at the genus level in female mice rather than in male mice (Fig. 3C). DNJ also specifically enhanced the abundance of *Akkermansia* (Fig. 3D and Table 1) in female mice, which was consistent with a previous report (24). Notably, the abundance of the butyrate-generating *Clostridium* XIVa was significantly decreased in the HFD + STZ-treated group and was substantially restored by DNJ supplementation in female mice but not in male animals (Fig. 3E and Table 1). Collectively, these results indicate that DNJ alters gut microbial structure in a sex-specific manner.

### Indole-3-propionic acid is positively associated with the antihyperlipidemic effect of DNJ.

**FIG 4:**
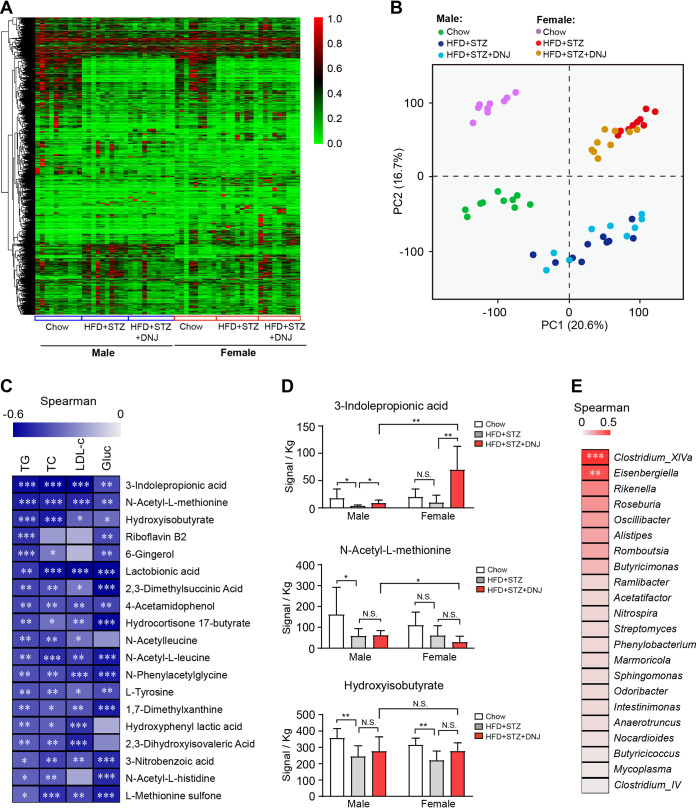
DNJ promotes indole-3-propionic acid (IPA) production in female mice. (A) Heatmap of fecal metabolite profile. (B) PCA plots revealing the separation and clustering of subjects in each group. (C) Heatmap of the correlations between 19 metabolites and serum lipid and glucose parameters among all groups. (D) Signal statistics of 3 fecal metabolites in each group. (E) Heatmap of the correlations between individual gut microbial genus and IPA level using samples from all groups. *N* = 10/9 in each male/female group, respectively. Data are presented as mean ± SEM. *, *P < *0.05; **, *P < *0.01; ***, *P < *0.001.

DNJ gender-specifically modulated the structure of live gut microbiota, we speculated that certain active metabolite(s) may play a key role in the antihyperlipidemic effect of DNJ. To address this, we performed metabolomic analysis on the fecal materials in mice by gas chromatography-time of flight mass spectrometry (GC-TOF/MS). Similarly to the analysis of live gut microbiota, the metabolomics also displayed an obvious gender disparity (Fig. 4A and B). Both male and female mice treated with HFD + STZ displayed distinct profiles of microbial metabolites from corresponding normal control mice (Fig. 4B). DNJ treatment markedly shifted the samples from the HFD + STZ group in female mice but not in male ones (Fig. 4B). Correlation analysis among individual metabolites and blood parameters revealed that 19 recognized metabolites were significantly negative to at least two serum parameters indicating hyperlipidemia or hyperglycemia (Fig. 4C). Among those, indole-3-propionic acid (IPA), *N*-acetyl-l-methionine, and hydroxyisobutyrate are the top 3 metabolites that are most closely related to the hyperlipidemia-alleviating role of DNJ (Fig. 4C). Detailed analysis of their abundance in each group revealed that neither feeding diet nor genders influenced *N*-acetyl-l-methionine and hydroxyisobutyrate, while IPA level was prominently elevated by DNJ in both male and female mice, with greater significance in latter. Importantly, the absolute abundance of IPA per kilogram in DNJ-treated female groups was much higher than that in male ones (69.5 versus 8.59; *P = *0.003) (Fig. 4D), indicating IPA to be the most potential metabolite involving in the beneficial effects of DNJ on hyperlipidemia. We further correlated the IPA content with each genus abundance to identify the main taxon that generates IPA. *Clostridium* XIVa and Eisenbergiella were the only two genera that are significantly positive to IPA content (Fig. 4E), implying that they may be the key gut bacteria that produce IPA.

### IPA possesses adequate lipid-lowering activities.

**FIG 5:**
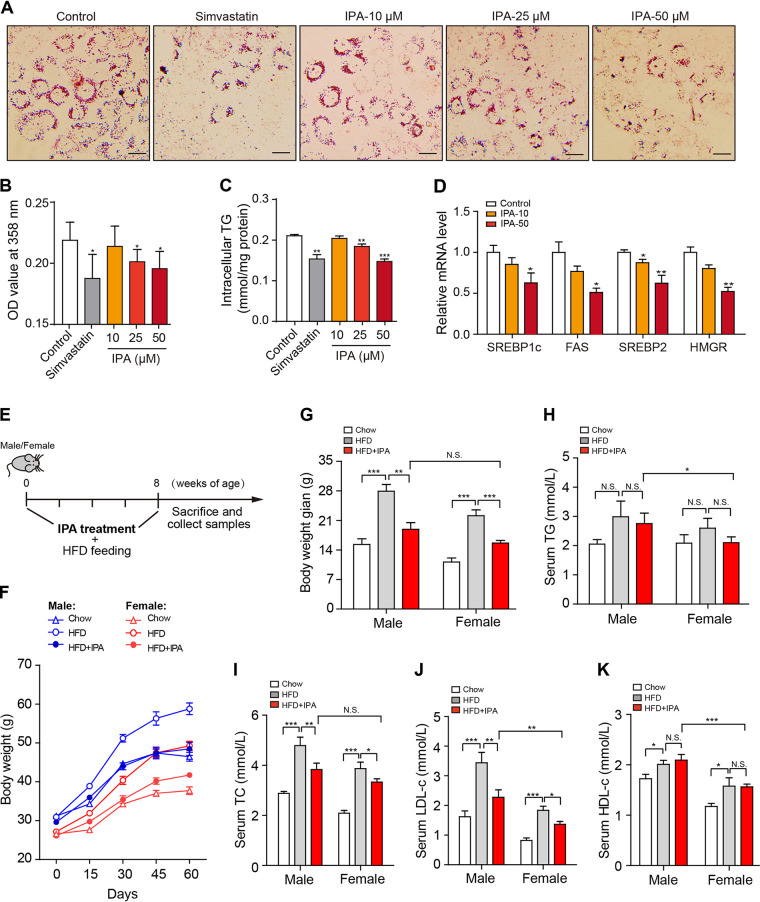
IPA attenuates hyperlipidemia. (A) Representative oil red O staining images of HepG2 cells. (B) Optical density at 358 nm (OD_358_) value after oil red O staining. (C) Intracellular TG content. (D) mRNA levels of key lipogenic genes. (E) Diagram of the animal experiment design. (F) Bodyweight curve. (G) Bodyweight gain. (H to K) Serum TC, TG, LDL-c, and HDL-c. *N* = 8 in each group. Data are presented as mean ± SEM. *, *P < *0.05; **, *P < *0.01; ***, *P < *0.001.

We then evaluated the lipid-lowering ability of IPA both *in vitro* and *in vivo*. Supplementation of oleic acid (OA; 100 μM) resulted in dramatically increased lipid accumulation in HepG2 cells. Treatment with IPA significantly diminished the OA-induced lipid accumulation in a dose-dependent manner, as revealed by oil red O staining (Fig. 5A and B) and intracellular TG quantification (Fig. 5C). Furthermore, IPA dose dependently decreased the transcription of the key genes involved in fatty acid (*SREBP1c* and *FAS*) and cholesterol biosynthesis (*SREBP2* and *HMGR*) (Fig. 5D). Further investigations in mice showed that oral administration of IPA significantly reduced the HFD-induced body weight gain in both male and female mice (Fig. 5E and F). IPA exhibited no impact on serum HDL-c level but markedly decreased serum levels of TC, LDL-c, and TG, exhibiting adequate antihyperlipidemic effects (Fig. 5G to J). Based on these results, we demonstrate that IPA is a potent lipid-lowering metabolite that may mediate the antihyperlipidemic effect of DNJ.

## DISCUSSION

As the major cause of cardiovascular disease (CVD), hyperlipidemia is prevalent around the world, with a higher incidence in women than men (1, 2). To date, therapeutic strategies specifically targeting the female population are yet inadequate, although many successful lipid-lowering drugs, such as statins and fibrates, are available for hyperlipidemia treatment (1, 2). However, whether statin therapy should be differentially prescribed according to sex is still a matter of debate (25), although CVD displays evident sex differences in clinical manifestation and therapeutic interventions. Thus, developing effective antihyperlipidemic drugs with gender specificity is more favorable to large numbers of female patients. Since drugs are lacking, we propose in our study that DNJ might be a potential agent against hyperlipidemia, mainly in the female population, due to its female-preferential beneficial effects on hyperlipidemia.

Mulberry leaves and its active component 1-deoxynojirimycin (DNJ) are well-documented functional food additives with confirmed antihyperglycemic effects (19, 20). Recent studies have revealed that DNJ is also an adequate antihyperlipidemic agent (22, 23). Review of the published papers about the lipid-modulatory action of DNJ showed that DNJ exerts a weak impact on blood LDL-c in male mice (26) but markedly decreased LDL-c in female animals (27), implying a potential female-preferential antihypercholesterolemic effect. We also demonstrated the female-specific cholesterol-lowering action of DNJ in mice fed an HFD (24). Given the dual effectiveness of DNJ in protecting against both hyperglycemia and hyperlipidemia, it should first be distinguished if the sex-specific activity of DNJ is specific for hyperlipidemia or applies to both. In this study, we treated animals with HFD feeding and a single small dose of STZ injection to get mild hyperglycemia combined with hyperlipidemia. Just as widely reported, treatment with DNJ dramatically alleviated hyperglycemia in both male and female mice, showing no sex disparities. In contrast, DNJ imposed no influence on serum levels of TC and LDL-c in male mice but significantly decreased their levels in female mice. DNJ was also more potent in alleviating hypertriglyceridemia and liver steatosis in female mice than in male ones. This obvious sex-specific hyperlipidemia-diminishing effect of DNJ is highly favorable for women with dyslipidemia uncontrolled by current medication.

Liver is the crucial organ to control levels of blood lipids. Three key steps, including lipid biosynthesis, transport, and degradation, work in coordination to determine the ultimate lipid pool (28). To illuminate the molecular mechanism by which DNJ suppresses hyperlipidemia, we evaluated regulations of DNJ on the transcription of hepatic genes involving in lipid biosynthesis (*SREBP1c*, *FAS*, *ACC*, *GPAT*, *SCD*, *ChREBP*, *SREBP2*, and *HMGR*), transport (*FATP*, *FABP*, *LXR*, and *CD36*) and oxidation (*CPT1*, *PPARα*, *AMPK*, and *ACO*). DNJ mainly reduced the expression of lipogenic genes, indicating that it may prevent the development of hyperlipidemia, at least in part, through inhibiting lipogenesis. In accordance, previous studies have proved that DNJ from various origins remarkably decreased mRNA levels of *FAS*, *ACC*, and *CEBPα* (27, 29). Concerning the other two steps, we did not observe significant influences of DNJ on the transcription of genes involved in lipid transport (except *CD36*) or oxidation, although some investigations have claimed that DNJ could also repress lipid uptake and stimulate lipid oxidation to exert lipid-lowering actions. For instance, Do et al. reported that DNJ isolated from Bacillus subtilis significantly decreased mRNA levels of *aP2* and *CD36* to improve hepatic lipid metabolism and mitochondrial function (27). *PPARα* and *AMPK* were also reported to be upregulated by DNJ treatment, leading to activation of the β-oxidation system in obese mice (22, 23). In our study, we exactly detected the inhibitory influence of DNJ on *CD36* in both male and female mice, which exhibiting no gender difference. In contrast, the expression of *PPAR*α and *AMPK* was subtly enhanced by DNJ treatment, possibly due to the various dosages used in different studies. As Wang et al. have reported, there is an obvious dose-dependent saddle-shaped regulation of DNJ on lipid metabolic gene expression in pigs (29). Nevertheless, our data present a common mechanism by which DNJ is capable of inhibiting expression of key lipogenic genes. Intriguingly, DNJ robustly downregulated genes involving in fatty acid biosynthesis in both male and female mice, yet with greater efficacy in the latter, which accords with the more apparent serum TG-lowering role of DNJ in female animals. Furthermore, treatment with DNJ significantly reduced mRNA levels of *SREBP2* and *HMGR*, two critical genes for cholesterol synthesis in female mice rather than in male ones, accounting for the female-specific beneficial effect of DNJ on blood TC and LDL-c levels. Therefore, it is plausible that DNJ possesses a sex-specific regulatory effect on lipid synthesis, especially for cholesterol, that improves hyperlipidemia in female mice.

Myriad evidence has demonstrated that gut microbiota plays critical roles in modulating lipid metabolism (30, 31). *Akkermansia* is a well-documented genus that prevents HFD-induced obesity, hyperglycemia, and hyperlipidemia (32–35). Another genus, the short-chain fatty acid (SCFA)-producing Clostridium spp. (e.g., *Clostridium* groups IV and XIVa) is effective at decreasing high levels of blood lipids, especially cholesterol and LDL-c, in animals and human beings (36, 37). Recently, several investigations have reported that DNJ or DNJ-enriched mulberry leaf extracts markedly altered gut microbial community to improve metabolic diseases by promoting the growth of *Akkermansia* spp. and SCFA-producing genera such as Bifidobacterium, Lactobacillus, and Alistipes (24, 38, 39). In particular, DNJ was reported to enrich the abundance of *Akkermansia* spp. in female mice (24), suggesting a close link of gut microbes with DNJ. According to 16S rRNA-based metatranscriptomics, we indeed observed the stimulatory modulation of DNJ on *Akkermansia* and *Clostridium* XIVa in female but not in male mice, consistent with the sex-specific antihyperlipidemic effect of DNJ. These results imply that *Akkermansia* and *Clostridium* XIVa may be the key gut bacteria responsible for DNJ’s efficacy in alleviating high blood lipids. As a matter of fact, 16S rRNA gene-based metagenomics was carried out prior to 16S rRNA metatranscriptomic sequencing, but the metagenomic analysis did not offer adequate insights into the regulation of DNJ on gut microbial community, even though a similar increasing trend of *Akkermansia* was observed. As for the reason, 16S rRNA gene pyrosequencing cannot distinguish live microbes and their dead bodies, so the influence of DNJ on live functional bacteria is masked. Hence, our results not only confirmed the sex-specific modulation of DNJ on certain key genera but also proved that 16S rRNA metatranscriptomic analysis is a more reliable analytic method for the gut microbial community.

Active metabolites are substances that directly mediate the pharmacological effect of functional microbes and their modulating agents (40, 41). Various types of gut microbial metabolites enable a decrease in blood lipids, for instance, SCFAs, especially propionate and butyrate, are common products of many polysaccharide-degrading bacteria, including the members belonging to *Clostridium* IV and XIVa groups (36, 37). Oral administration of SCFAs effectively prevents diet-induced hyperlipidemia, particularly for hypercholesterolemia (42, 43). Indole derivatives, another kind of active metabolite, exhibit potent anti-inflammatory and lipid-lowering effects *in vivo* and *in vitro* (44–47). We further performed a metabolomics analysis on mouse feces to discover the potential active metabolites. As expected, fecal metabolomics also displayed obvious gender disparity, and 19 compounds were significantly negatively related to blood TC, TG, LDL-c, and glucose. Of note, the bodyweight of mice is a critical factor for statistical analysis. Given that, we found indole-3-propionic acid (IPA) was the top related metabolite and was only remarkably elevated by DNJ in both female and male mice, yet with more significance in the former. The absolute abundance of IPA per kilogram in female mice treated with DNJ was approximately 8-fold larger than that in male ones (female DNJ group versus male DNJ group: 69.5 versus 8.59). Although DNJ significantly increased IPA content in male mice, the absolute amount of IPA in the male HFD + DNJ group was only comparable to that in the female HFD-alone group, and thus DNJ still cannot exert an obvious lipid-lowering effect in male mice. These results definitely support our conclusion that the prominent and robust increase of IPA may contribute to the antihypercholesterolemic effect of DNJ in female mice. Furthermore, both *in vitro* and *in vivo* experiments showed that IPA significantly diminished OA- and HFD-induced lipid elevation accompanied by the reduced transcription of *SREBP1c* and *FAS*, thus corroborating the notion that IPA functions as the key microbial metabolite responsible for the protective role of DNJ on hyperlipidemia.

Another important finding is that *Clostridium* XIVa taxa were significantly positively associated with IPA production by correlation analysis of IPA content and genus abundance in each sample, suggesting them as the candidate bacteria for IPA generation. Further investigations are awaited to verify this interesting conjecture. Due to the limitation of 16S rRNA pyrosequencing, we cannot identify the specific species that may produce IPA. However, Clostridium sporogenes is the only proved intestinal bacteria to produce IPA (32). Given the elevated growth of *Clostridium* XIVa induced by DNJ and its positive correlation with IPA content, we conclude that DNJ female specifically alters the composition of the gut microbiota, characterized by the enrichment of certain members in *Clostridium* XIVa, to stimulate IPA generation, ultimately ameliorating hyperlipidemia.

**FIG 6:**
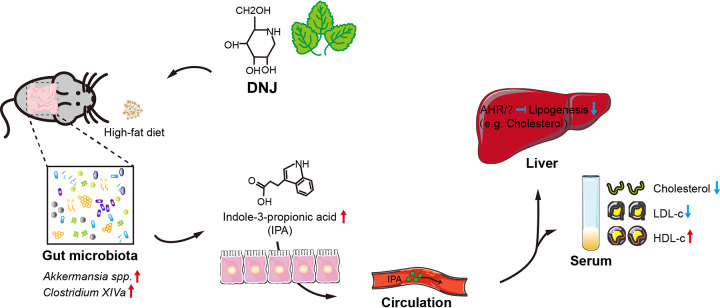
Schematic diagram illustrating a potential mechanism for DNJ to exert a sex-specific antihyperlipidemic effect. DNJ sex-specifically modulates gut microbiota and promotes gut microbes such as *Clostridium* XIVa spp. to produce indole-3-propionic acid (IPA) in female mice. The increased circulation level of IPA results in the downregulation of hepatic lipogenic genes and ultimately in decreased serum level of lipids, especially cholesterol and low-density lipoprotein cholesterol.

Notably, indole derivatives (e.g., indole-3-acetic acid and indole-3-propionic acid) usually act as ligands of the aryl hydrocarbon receptor (AHR), which is emerging as a crucial factor to regulate lipid metabolism and intestinal inflammation (46, 48, 49). Once activated, AHR exhibits remarkably negative regulation on the expression of *SREBP1c* and *FAS* to attenuate lipid accumulation in the liver. Together with our findings, we propose that IPA, as the key microbial metabolite, mediates the antihyperlipidemic effect of DNJ, possibly following the IPA-AHR-lipid metabolism axis. Collectively, a fat-rich diet leads to hyperlipidemia and pronounced dysbiosis. The beneficial microbes and related microbial metabolite indole-3-propionic acid (IPA) are consequently decreased. Oral administration of DNJ restores the dysregulated gut microbial community, elevates the abundances of certain gut bacteria with lipid-lowering effects (e.g., *Akkermansia* spp. and *Clostridium* XIVa), and stimulates the generation of IPA, which is capable of inhibiting the transcription of hepatic lipogenic genes, and ultimately improves hyperlipidemia-related symptoms, including body weight, TC, TG, and LDL-c (Fig. 6).

### Conclusion.

In summary, our study has demonstrated the mulberry leaf-derived DNJ is a sex-specific antihyperlipidemic agent that preferentially ameliorates hypercholesterolemia in females. The sex-specific modulation of gut microbes and their active metabolite, indole-3-propionic acid (IPA), plays a key role in the gender-differential therapeutic effect of DNJ, which suggests that the gender disparities in regulating the gut microbiota may provide a novel strategy for the development of next-generation antihyperlipidemic drugs.

## MATERIALS AND METHODS

### Mulberry leaves and DNJ preparation.

Fresh mulberry leaves (Morus multicaulis Perr.) were collected at a mulberry farm in July in Chun’an County, Zhejiang Province, China. The leaves were washed with clean water, dried at 50°C for 36 h, and then ground into powder. The leaf powder was extracted with 50% ethanol, concentrated by rotary evaporation, and suspended in carboxymethyl cellulose (CMC)-Na 1% to form a homogenous solution.

DNJ was extracted from mulberry leaves and purified by a liquid chromatography-mass spectrometry (LC-MS) system (Waters, USA) as previously reported (24). The purity of DNJ was confirmed to be over 95% by high-performance liquid chromatography (HPLC) analysis. The yield of DNJ was about 0.3% wet weight of mulberry leaves.

### Diets.

A standard rodent chow consisting of 4.5% fat, 50% carbohydrate, and 22% protein with a total calorific value of 6.095 kcal/kg and a high-fat diet (HFD) consisting of 22% fat, 35% carbohydrate, and 21% protein with a total calorific value of 10.516 kcal/kg were obtained from Shanghai SLAC Laboratory Animal Co., Ltd.

### Animal experiment.

All the animal experiments were performed following the National Institutes of Health guide for the care and use of Laboratory Animals (NIH Publications No. 8023, revised 1978) and were approved by the Medical Ethics Committee of Zhejiang Academy of Agricultural Sciences (No. ZAAS-20170812011).

**(i) MLE function *in vivo*.** Male and female ICR mice (40 each), 8 weeks old, with a bodyweight of 16 to 20 g, were purchased from Shanghai SLAC Laboratory Animal Co., Ltd. (Shanghai, China). The animals were randomly divided based on sex into 8 groups (male and female subgroups: chow, HFD, HFD-STZ-MLE200, and HFD-STZ-MLE400) with 10 mice in each group. The chow group was fed with standard rodent chow diet, while the other groups were given a high-fat diet (HFD) for 8 weeks, followed by a single intraperitoneal (i.p.) injection (30 mg/kg) of streptozotocin (STZ, V900890; Merck) dissolved in 0.1 M cold citrate buffer (pH 4.5) to establish mouse models suffering both hyperlipidemia and hyperglycemia. After STZ treatment for 15 days, the MLE groups were administered MLE (200 or 400 mg/kg) daily by oral gavage, while the STZ group (as a negative control) and the chow group (as a normal control) were given an equal volume of distilled water. During the 12-week experimental period, bodyweight was recorded weekly. At the end of the experiment, animals fasted overnight, and blood samples were collected for estimation of serum levels of glucose, total triglyceride (TG), total cholesterol (TC), low-density lipoprotein cholesterol (LDL-c), and high-density lipoprotein cholesterol (HDL-c) by respective kits (Nanjing Jiancheng Bioengineering Institute).

**(ii) DNJ function *in vivo*.** For DNJ evaluation, 30 male and 27 female ICR mice, 8 weeks old, with a bodyweight of 16 to 20 g, were randomly divided based on sex into 6 groups with 10 and 9 mice in each male and female group, respectively. The chow group was fed with standard rodent chow diet, while other groups were given an HFD for 8 weeks, followed by a single intraperitoneal injection (30 mg/kg) of STZ. After STZ treatment for 15 days, the DNJ groups were administered DNJ (50 mg/kg) daily by oral gavage, while the STZ group (as a negative control) and the chow group (as a normal control) were given an equal volume of distilled water. During the 12-week experimental period, bodyweight was recorded weekly, and blood lipids and glucose levels were measured from orbital venous blood at the 4th, 8th, and 12th weeks. Stools for phylogenetic analysis were collected on the day before animals were sacrificed. At the end of the experiment, animals fasted overnight, and blood samples were collected for estimation of serum levels of total triglyceride (TG), total cholesterol (TC), low-density lipoprotein cholesterol (LDL-c), and high-density lipoprotein cholesterol (HDL-c) by respective kits (Nanjing Jiancheng Bioengineering Institute). Simultaneously, liver tissues were taken for the subsequent histological analysis.

**(iii) IPA function *in vivo*.** To assess the antihyperlipidemic effect of the active metabolite, indole-3-propionic acid (IPA, catalog no. I103959; Aladdin), 24 male (28 to 32 g) and 24 female ICR mice (25 to 30 g), were randomly divided into 6 groups with 8 animals per group. The normal (chow) group fed on standard rodent chow diet, while other groups were fed with an HFD for 8 weeks. The chow and HFD groups were given an equal volume of distilled water, while the HFD + IPA group was orally administered IPA (100 mg/kg). Bodyweight was recorded weekly. At the end of the experiment, animals fasted overnight, and blood samples were collected for estimation of serum TG, TC, LDL-c, and HDL-c.

### Quantitative real-time PCR analysis.

Liver samples were collected in all animal experiments for quantitative real-time PCR (qRT-PCR) to analyze hepatic gene expression. Total RNA was extracted with TRIzol reagent (catalog no. 15596018; Thermo Fisher), and cDNA was synthesized from 2 mg RNA with random primers using a reverse transcription kit (catalog no. 11121ES60; Yeasen Biotechnology). qRT-PCRs were performed with SYBR green PCR mix (A25742; Thermo Fisher) in a StepOnePlus real-time PCR system (Applied Biosystems). β-Actin was used as an internal control. All primer sequences used in the study are provided in Table S1 in the supplemental material.

10.1128/mSystems.00313-20.2 TABLE S1 Primers used for quantitative real-time PCR (qRT-PCR) in this study. Download Table S1, DOC file, 0.04 MB. Copyright © 2020 Li et al. 2020 Li et al. This content is distributed under the terms of the Creative Commons Attribution 4.0 International license.

### Cell culture and treatment.

The HepG2 cell line was cultured in Dulbecco’s modified Eagle’s medium (DMEM, catalog no. 10566-016; Thermo Fisher) supplemented with 10% fetal bovine serum (catalog no. 10099141; Thermo Fisher) at 37°C in a humidified atmosphere consisting of 5% CO_2_. Before the examination, oleic acid (OA, 100 μM) combined with 3-indolepropionic acid (10, 25, and 50 μM, catalog no. S4809; Selleck) or simvastatin (10 μM, catalog no. S1792; Selleck) were supplemented into DMEM overnight. Cells were stained with oil red O solution (catalog no. G1262; Solarbio) following the manufacturer’s instructions. For further quantification, optical density (OD) values of cells after treatment were measured at 358 nm using a microplate reader (BioTek, USA). The amount of intracellular TG was determined using a TG kit (catalog no. BC0625; Solarbio).

### Microbial DNA and mRNA extraction.

Before sacrifice, fecal samples were collected from mice used in DNJ experiments and then snap-frozen in liquid nitrogen and stored at −80°C. Microbial DNA and total mRNA were extracted from fecal materials using the QIAamp DNA Stool minikit (catalog no. DXT-51504; Qiagen) and the Qiagen RNeasy minikit (catalog no. DXT-74106; Qiagen), respectively. Then, the samples underwent 16S rRNA metagenomic/metatranscriptomic sequencing at the V4-V5 regions on an Illumina HiSeq 2500 instrument according to the manufacturer’s instructions.

### Bioinformatics.

Fast Length Adjustment of SHort reads (FLASH) was used to merge paired-end reads from 16S rRNA metagenomic/metatranscriptomic sequencing. Low-quality reads were filtered and chimera reads were removed by USEARCH (64-bit v8.0.1517). Operational taxonomic units (OTUs) were aligned by the UCLUST algorithm with 97% identity and taxonomically classified using the SILVA 16S rRNA database v128. Alpha and beta diversities were generated using Quantitative Insights Into Microbial Ecology (QIIME). Principal-component analysis (PCA) and principal-coordinate analysis (PCoA) were performed using the *vegan* package in R.

### Metabolomics analysis.

Fecal samples were collected from each mouse in the DNJ function evaluation assay for subsequent metabolomics analysis. Untargeted metabolomics profiling was performed on the XploreMET platform (Metabo-Profile, Shanghai, China). The sample preparation procedures are referred to in their previously published methods and were performed with minor modifications. More details are provided in the supplemental material.

### Statistical analysis.

All data presented in this paper are shown as mean ± standard error of the mean (SEM). For pharmacological results, comparisons between groups were assessed by one-way analysis of variance (ANOVA) with Dunnett’s test as a *post hoc* test. A *P* value of <0.05 was considered statistically significant. For phylotype analysis, the alpha diversity of the microbiome was calculated based on the OTU level using mothur (version 1.30.1). Principal-coordinate analysis (PCoA) and nonmetric multidimensional scaling (NMDS) analysis were performed using R and visualized by the R package, and significant differences were evaluated by adonis analysis. Other significant differences were assessed using the Mann-Whitney U test or unpaired Student’s *t* test in R. *P* values were adjusted for multiple comparisons using the Benjamini and Hochberg correction. False discovery rate and significance were set at *q* < 0.05.

### Data availability.

NCBI SRA BioProject accession number PRJNA633670 includes all raw data of 16S rRNA gene and rRNA sequencing. All sequencing data involved in this work can be acquired at NCBI.

10.1128/mSystems.00313-20.1 TEXT S1 Metabonomics analysis. Download Text S1, DOC file, 0.03 MB. Copyright © 2020 Li et al. 2020 Li et al. This content is distributed under the terms of the Creative Commons Attribution 4.0 International license.
