# Supplementary figures and images for: The Gut Microbiota-Produced Indole-3-Propionic Acid Confers the Antihyperlipidemic Effect of Mulberry-Derived 1-Deoxynojirimycin
Source: mSystems. 2020 Oct 6;5(5):e00313-20. doi: 10.1128/mSystems.00313-20 (PMC7542557; doi:10.1128/mSystems.00313-20)

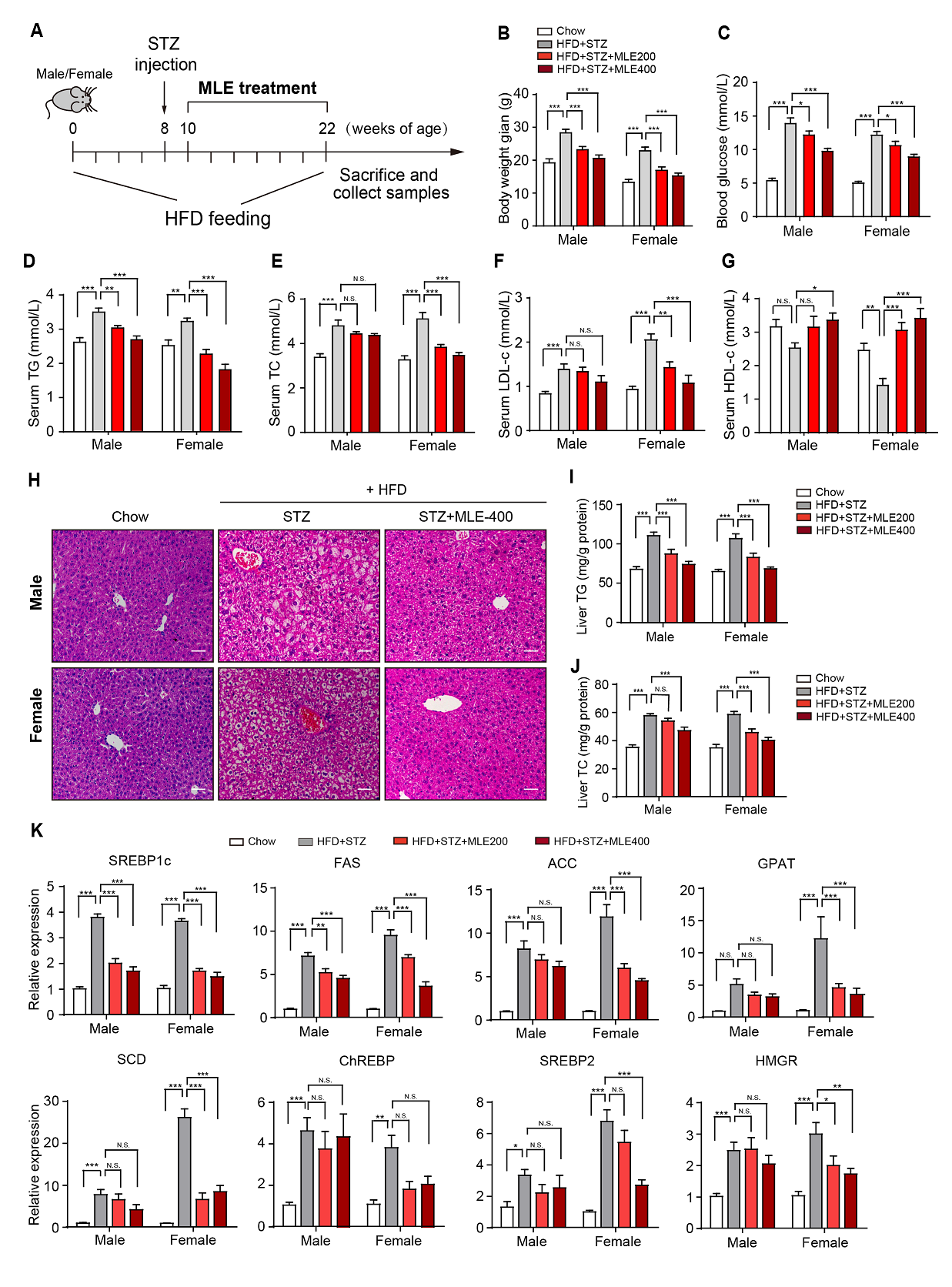

Supplement: FIG S1 [file mSystems.00313-20-sf001.tif]

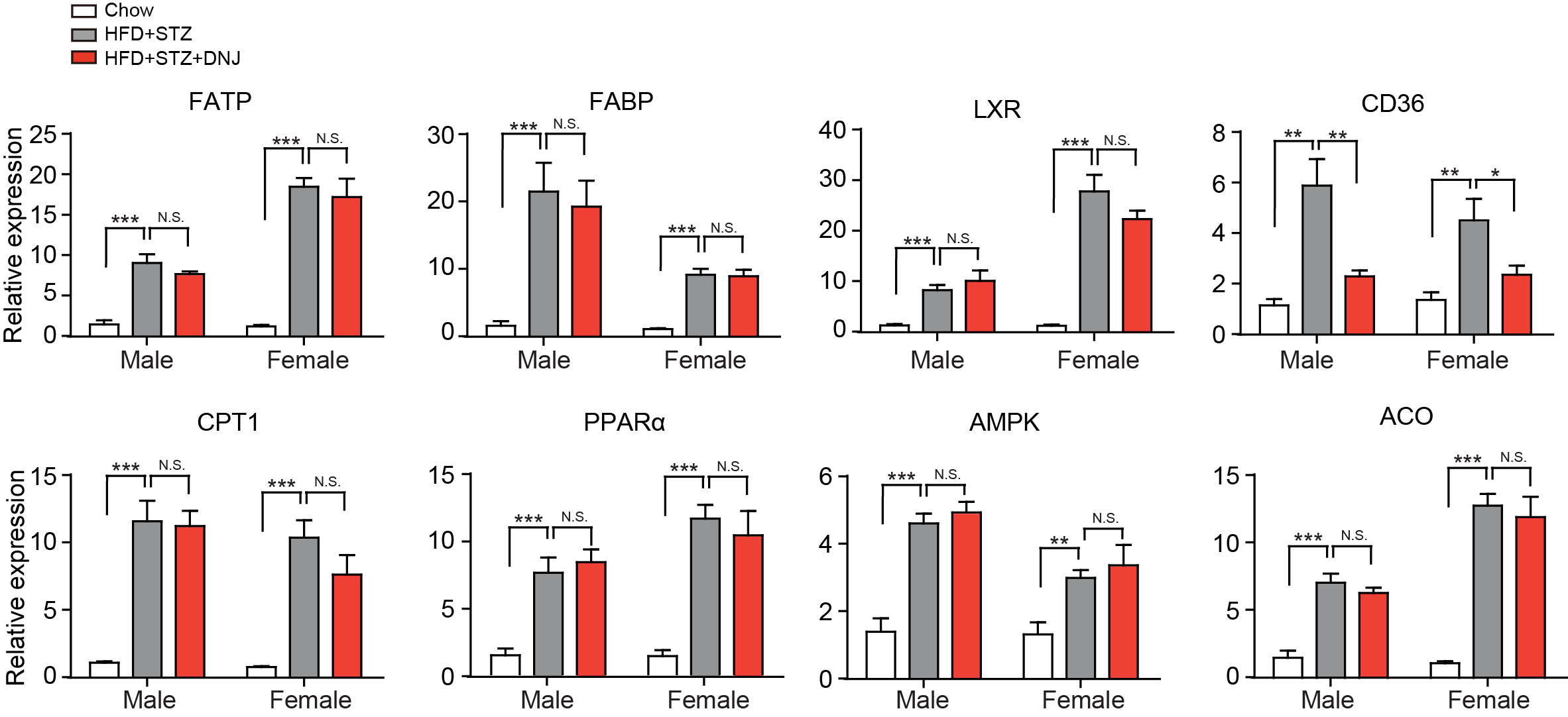

Supplement: FIG S2 [file mSystems.00313-20-sf002.tif]

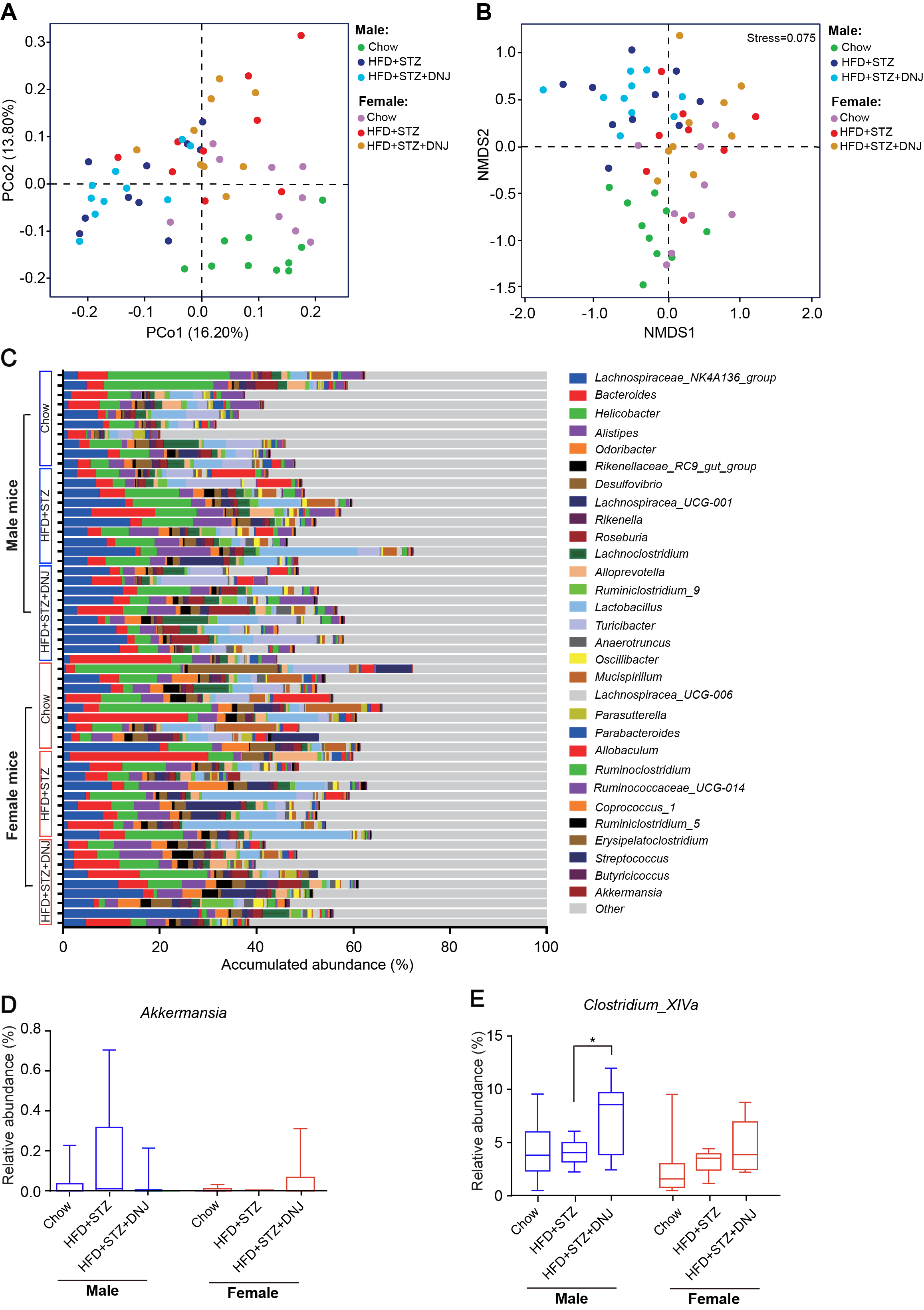

Supplement: FIG S3 [file mSystems.00313-20-sf003.tif]
