# Supplementary material for: The Gut Microbiota-Produced Indole-3-Propionic Acid Confers the Antihyperlipidemic Effect of Mulberry-Derived 1-Deoxynojirimycin
Source: mSystems. 2020 Oct 6;5(5):e00313-20. doi: 10.1128/mSystems.00313-20 (PMC7542557; doi:10.1128/mSystems.00313-20)
Supplement: TABLE S1 [file mSystems.00313-20-st001.doc]

**TABLE S1** Primers used for qRT-PCR in this study

| **Gene** | **Forward primer (5’-3’)** | **Reverse primers (5’-3’)** |
| --- | --- | --- |
| SREBP1c | GGAGATGTTCACGGAATAGCC | GTTTCTTTGTGGACGGGGACT |
| FAS | TCTTCTGGCTGTGAACACTGT | AGCAACCATAGGCGATTTCT |
| ACC | CCTTCTTGATGTTAGGAGGCTT | ACCAGCACCGAGACTGAACT |
| GPAT | GTGGACAAAGATGGCAGCAGA | CAGAACAGCAGTGGGACAAGA |
| SCD | GCAAGAAGGTGCTAACGAACA | GAGAAGGGCGGAAAACTGGAC |
| ChREBP | CCAGCCTCAAGGTGAGCAAA | CATGTCCCGCATCTGGTCA |
| SREBP2 | TCCAGAAGATGACAGACCTCA | GGTGGGACTTTCCTGCTAAT |
| HMGR | AGCTTGCCCGAATTGTATGTG | TCTGTTGTGAACCATGTGACTTC |
| FATP | CTGGGACTTCCGTGGACCT | TCTTGCAGACGATACGCAGAA |
| FABP | TTCCTGTCGTCTGCGGTGATT | GCGTAAATGGGGATTTGGTCA |
| CD36 | CCCAGTCTCATTTAGCCACAG | CATTTCCTACATGCAAGTCCA |
| CPT1 | CGGCTATGGTGTTTCCTACA | TTGTCTCAAGTGCTTCCCAA |
| PPARα | TATTGAGCCGATACCAGCAT | CCTTCTGTGTTCACCCTGATT |
| AMPK | CTGTATTCCCGCTGTGTAGAA | TCCTATTTAGTTGGCTCCTCCT |
| ACO | AATGCTGTCACCCAGGAGTT | GAGCCCTCACCATAGTTCTCA |
| β-actin | CATGTACGTTGCTATCCAGGC | CTCCTTAATGTCACGCACGAT |
