# Supplementary material for: The Gut Microbiota-Produced Indole-3-Propionic Acid Confers the Antihyperlipidemic Effect of Mulberry-Derived 1-Deoxynojirimycin
Source: mSystems. 2020 Oct 6;5(5):e00313-20. doi: 10.1128/mSystems.00313-20 (PMC7542557; doi:10.1128/mSystems.00313-20)
Supplement: TEXT S1 [file mSystems.00313-20-s0001.doc]

**TEXT 1** Metabonomics analysis

The untargeted metabolomics profiling was performed on XploreMET platform (Metabo-Profile, Shanghai, China).

*Sample preparation:* The frozen samples were kept on a dry ice ethanol bath. About 50 mg material was accurately weighed in an Eppendorf Safelock microcentrifuge tube, to which 25 mg of pre-chilled zirconium oxide beads, 10 μl of internal standard, and 50 μl of 50% pre-chilled methanol were added for automated homogenization (BB24, Next Advance, Inc., Averill Park, NY, USA). After centrifugation at 14,000 g and 4 ^o^C for 20 min (Microfuge 20R, Backman Coulter, Inc., Indianapolis, IN, USA), the supernatant was carefully transferred to an autosampler vial (Agilent Technologies, Foster City, CA, USA). Each aliquot of 175 μl of pre-chilled methanol/chloroform (v/v=3/1) was added to the residue for the second extraction. After centrifugation at 14,000 g and ^o^C for 20 min, the supernatant was carefully transferred to an autosampler vial. All samples in autosampler vials were evaporated briefly to remove chloroform using a CentriVap vacuum concentrator (Labconco, Kansas City, MO, USA), and further lyophilized with a FreeZone freeze dryer equipped with a stopping tray dryer (Labconco, Kansas City, MO, USA). The sample derivatization and injection were performed by a robotic multipurpose sample MPS2 with dual heads (Gerstel, Muehlheim, Germany). Briefly, the dried sample was derivatized with 50 μl of methoxyamine (20mg/mL in pyridine) at 30 ^o^C for 2h, followed by the addition of 50 μl of MSTFA (1% TMCS) containing FAMEs as retention indices at 37.5 ^o^C for another 1h using the sample preparation head. In parallel, the derivatized samples were injected with sample injection head after derivatization.

*Instrumentation*: A time-of-flight mass spectrometry (GC-TOF/MS) system (Pegasus HT, Leco Corp., St. Joseph, MO, USA) with an Agilent 7890B gas chromatography and a Gerstel multipurpose sample MPS2 with dual heads (Gerstel, Muehlheim, Germany). A Rxi-5 ms capillary column (30m x 250 μm i.d., 0.25 μm film thickness; Restek corporation, Bellefonte, PA, USA) was used for separation. Helium was used as the carrier gas at a constant flow rate of 1.0 mL/min. The temperature of the injection and transfer interface were both set to 270 ^o^C. The source temperature was 220 ^o^C. The measurements were made using electron impact ionization (70 eV) in the full scan mode (m/z 50-500). The detailed instrument settings are briefly described in Supplementary Table S5. Instrument optimization was performed every 24 hours.

*Metabolite annotation*: Metabolite annotation was performed by comparing the retention indices and mass spectral data with those previously generated from reference standards of known structures present in Jia Lib metabolite database using our proprietary software XploreMET. The current Jia Lib comprised over 1,200 mammalian metabolites with chemicals present in Jia Lib were commercially purchased from Sigma-Aldrich (St. Louis, MO, USA), Santa Cruz (Dallas, TX, USA), Nu-chek Prep (Elysian, MN, USA), and synthesized in the laboratory.

*Data analysis*: The raw data generated by GC-TOF/MS were processed using XploreMET for automated baseline denoising and smoothing, peak picking, and deconvolution, creating a reference database from the pooled QC samples, metabolite signal alignment, missing value correction and imputation, and QC correction. Each data set was transformed into comparable data vectors for statistical analysis. All measurements were mean-centered and scaled by the standard deviation of the observed measurements.
